# Flight-related determinants of healthcare services utilization of asylum seekers and refugees in Germany: a study based on the German Socio-Economic Panel

**DOI:** 10.1093/eurpub/ckae135

**Published:** 2024-08-30

**Authors:** Thomas Grochtdreis, Hans-Helmut König, Judith Dams

**Affiliations:** Department of Health Economics and Health Services Research, Hamburg Center for Health Economics, University Medical Center Hamburg-Eppendorf, Hamburg, Germany; Department of Health Economics and Health Services Research, Hamburg Center for Health Economics, University Medical Center Hamburg-Eppendorf, Hamburg, Germany; Department of Health Economics and Health Services Research, Hamburg Center for Health Economics, University Medical Center Hamburg-Eppendorf, Hamburg, Germany

## Abstract

The aim of this study was to analyze the associations between healthcare services utilization and flight-related characteristics of asylum seekers and refugees in Germany. The 2020 wave of the German Socio-Economic Panel’s Survey of Refugees was used to compile a sample of asylum seekers and refugees (*n* = 3134). Healthcare services utilization was measured using the self-reported number of visits to primary care physicians and hospitalization. Only the feeling of being welcome and worries about not being able to stay in Germany were identified as potential flight-related determinants of healthcare services utilization.

## Introduction

During the European refugee and displacement crisis after 2015, Germany has played a key role as a receiving country along with Italy, France, and Sweden, with ∼1.8 million asylum seekers and refugees (ASRs) registered in the German Central Register of Foreign Nationals [1]. In Germany, asylum seekers in particular are subject to certain effects of legal regulations resulting from their residence status, such as restricted access to the labor market, no settlement permission, and the receipt of reduced social benefits [2]. These structural restrictions are known to have an impact on health and healthcare services utilization among asylum seekers and may affect refugees.

In addition, migration background and migration-related characteristics, such as the reason for migration, cultural barriers, and language skills, can be predisposing determinants of healthcare services utilization among ASRs [3–5]. Compared with non-migrants, persons with a migration background in Germany utilized specialists in outpatient care less frequently, while evidence on utilization of primary care physicians (PCPs) and hospitalization was inconclusive [6]. Among persons with a direct migration background in Germany, the country of birth, connectedness with the country of birth, and oral German language skills were identified as possible determinants of PCPs utilization [4].

However, apart from the general structural determinants of health and healthcare services utilization of ASRs, such as their exclusion from the welfare system and their living situation in the receiving country [2], individual predisposing flight-related determinants of healthcare services utilization are unknown among ASRs who arrived in a European country after 2015. The aim of this study was therefore to analyze the associations between healthcare services utilization and flight-related characteristics of ASRs in Germany.

## Methods

### Sample

The sample was compiled from the Survey of Refugees, which was integrated into the German Socio-Economic Panel (SOEP) [7]. By 2024, the Survey of Refugees consisted of three subsamples (M3 to M5) with six consecutive waves (33–38), but only wave 37 in 2020 contained data on healthcare services utilization. After restricting the sample to persons who provided information on healthcare services utilization, age, and country of birth, the analysis sample consisted of *n* = 3134 adult persons.

### Measures

In the SOEP, healthcare services utilization was measured by the self-reported number of visits to PCPs in the previous 3 months and self-reported hospitalization within 1 year. Furthermore, self-reported pre-flight-related characteristics, post-flight-related characteristics, sociodemographic characteristics, and current health, measured on a five-point Likert scale from very good to very poor, were derived from the SOEP.

### Statistical analysis

Multiple imputation by chained equations with predictive mean matching (*m* = 20 imputations) was used to handle missing values. Descriptive statistics of flight-related and sociodemographic characteristics of the sample were calculated.

Associations between the number of visits to PCPs and flight-related characteristics were examined using a generalized linear model with gamma family and log link function. Associations between hospitalization and flight-related characteristics were examined using a logistic regression model. Current health status, sociodemographic characteristics, and the subsample were included as covariates in both models.

## Results

### Sample characteristics

Approximately half (52%) of the total sample (*n* = 3134) was female, with a mean age of 37 years and the majority (61%) aged between 25 and 44 years. The majority of the ASRs were born in Syria (62%), Iraq (13%), and Afghanistan (10%). The current health of the ASRs was generally (very) good (75%). The sociodemographic and flight-related characteristics of the sample are shown in Tables S1 and S2 in the online supplementary material.

### Number of visits to PCPs

The mean number of visits to PCPs in the previous 3 months was 1.45 [standard error (SE) 0.10], with 51% of all ASRs having visited a PCP at least once. Among those who had visited a PCP (*n* = 1597), the mean number of PCP visits was 2.84 (SE 0.10).

The possession of a temporary residence permission was associated with a higher number of visits to PCPs (−20%, *P* = .039) compared with no residence permission. Feeling barely or never welcome was associated with a higher number of visits to PCPs than feeling fully or predominantly welcome in Germany (+48%, *P *=* *.039). Great worries about not being able to stay in Germany were associated with a 15% lower number of visits to PCPs compared with no worries (*P *=* *.023; Table 1).

**Table 1.** Model of number of visits to primary care physicians in the previous 3 months, and model of hospitalization within 1 year (*n* = 3134)

Variable	Model 1^a^ (dependent variable number of visits to PCPs in the previous 3 months)		Model 2^b^ (dependent variable hospitalization within 1 year)	
	Exp(b) (SE)	*P*-value	OR (SE)	*P*-value
Current health status (Ref. very good)				
Good	1.65 (0.12)	<.001	1.64 (0.25)	.001
Satisfactory	3.42 (0.29)	<.001	3.35 (0.58)	<.001
Poor	6.43 (0.75)	<.001	6.60 (1.29)	<.001
Very poor	8.04 (1.08)	<.001	6.94 (1.95)	<.001
Gender (Ref. male)				
Female	1.01 (0.06)	.916	1.17 (0.13)	.147
Age (years)	1.00 (0.00)	.344	0.99 (0.01)	.039
Country of birth (Ref. Syria)				
Iraq	1.01 (0.10)	.881	1.16 (0.21)	.411
Afghanistan	0.77 (0.08)	.013	1.10 (0.23)	.642
Europe	0.67 (0.16)	.094	1.08 (0.46)	.847
Africa	0.73 (0.10)	.018	1.09 (0.27)	.731
Other Asia^c^	0.86 (0.12)	.277	1.08 (0.29)	.787
School-leaving qualification^d^ (Ref. Secondary general school)				
Secondary school	1.17 (0.10)	.062	1.09 (0.17)	.565
Academic secondary school	1.01 (0.07)	.896	1.02 (0.14)	.902
No school-leaving qualification	1.41 (0.26)	.060	1.13 (0.43)	.755
Employment (Ref. unemployed)				
Employed	0.76 (0.05)	<.001	0.72 (0.09)	.013
Religious affiliation (Ref. other faith or non-denominational)				
Muslim	1.00 (0.08)	.970	1.00 (0.16)	.978
Oral ability in the German language (Ref. very good)				
Good	0.88 (0.09)	.196	0.68 (0.14)	.066
Not bad	0.79 (0.09)	.032	0.91 (0.19)	.643
Fairly bad	0.81 (0.10)	.085	0.88 (0.21)	.588
Not at all	1.05 (0.22)	.831	1.64 (0.63)	.203
Economic situation in country of origin (Ref. average)				
Above average	0.93 (0.06)	.287	1.03 (0.14)	.841
Below average	0.94 (0.08)	.566	0.88 (0.13)	.401
Connectedness with country of origin (Ref. in some respects)				
(Very) strong	1.05 (0.07)	.471	0.89 (0.12)	.376
Hardly or not at all	1.04 (0.09)	.632	0.97 (0.15)	.854
Reason for leaving country of origin (Ref. no/not applicable)^e^				
Fear of violent conflict/war	0.90 (0.08)	.251	1.13 (0.20)	.473
Persecution	0.97 (0.06)	.597	0.84 (0.11)	.176
Discrimination	0.99 (0.07)	.826	0.88 (0.12)	.330
Living conditions	1.11 (0.09)	.163	1.25 (0.18)	.130
Economic situation	0.90 (0.07)	.204	1.04 (0.16)	.777
Residence status^f^ (Ref. no residence permission^g^)				
Temporary residence permission^h^	0.80 (0.09)	.039	0.87 (0.18)	.522
Permanent residence permission^i^	1.00 (0.19)	.999	0.51 (0.22)	.110
Satisfaction with living situation (Ref. neither satisfied nor dissatisfied)				
Satisfied	1.03 (0.08)	.671	0.96 (0.13)	.763
Dissatisfied	1.18 (0.11)	.078	0.94 (0.16)	.728
Discrimination due to origin (Ref. never)				
Rarely	1.10 (0.07)	.165	1.00 (0.12)	.980
Often	0.97 (0.12)	.837	0.88 (0.25)	.661
Feeling of being welcome (Ref. fully or predominantly)				
In some respects	0.98 (0.09)	.833	1.45 (0.25)	.031
Barely or never	1.48 (0.28)	.039	0.58 (0.21)	.128
Feeling of missing people from the country of origin (Ref. rarely or never)				
Sometimes	0.92 (0.09)	.411	0.90 (0.17)	.600
(Very) often	1.01 (0.09)	.912	0.98 (0.17)	.912
Worries of not being able to stay in Germany (Ref. no worries)				
Some worries	1.10 (0.09)	.211	0.88 (0.13)	.400
Great worries	0.85 (0.06)	.023	0.83 (0.11)	.167
Worries of not being able to return to country of origin (Ref. no worries)				
Some worries	0.92 (0.06)	.262	0.87 (0.13)	.345
Great worries	0.99 (0.09)	.867	1.20 (0.21)	.307
Subsample	✓		✓	
Constant	1.23 (0.32)	.427	0.16 (0.07)	<.001

PCP, primary care physician, SE, standard error, OR, Odds ratio.

a Generalized linear model with gamma family and log-link function and robust standard errors;

b Logistic regression model with robust standard errors.

c Without Syria, Iraq, and Afghanistan.

d ‘Other school-leaving qualification’ is not shown.

e Multiple answers possible.

f Another/no residence status not shown.

g Permission to stay pursuant to Section 55 of the German Asylum Law (asylum seekers) or temporary suspension of deportation according to Section 60a of the German Residence Act.

h Settlement permit according to Section 26 sub-section 3 of the German Residence Act.

i Residence permit according to Section 25 sub-section 1 of the German Residence Act (persons entitled to asylum), according to Section 25 sub-section 2 of the German Residence Act (persons with refugee status), according to Section 22 or Section 23 of the German Residence Act (admission on humanitarian grounds), or residence permit pursuant to § 23a or § 25 sub-section 3, 4, or 5 of the German Residence Act (admission on other humanitarian grounds).

### Hospitalization

Of all ASRs, 14% (*n* = 430) were hospitalized within 1 year. Among those who were hospitalized, the mean number of nights spent in hospital was 7.34 (SE 0.66), whereas the mean number of nights spent in hospital for the whole sample was 1.01 (SE 0.10).

Feeling welcome only in some respects was associated with a higher odds of hospitalization compared with feeling fully or predominantly welcome in Germany (odds ratio 1.45, *P *=* *.031; Table 1).

## Discussion

A feeling of being welcome and worries about not being able to stay in Germany were identified as flight-related determinants of healthcare services utilization. Feeling less welcome in Germany was not only associated with a higher utilization of PCPs and hospitalization, but also with a lower mental health-related quality of life among ASRs in Germany [8]. Greater worries about not being able to stay in Germany were associated with a lower utilization of PCPs, but also with a lower mental health-related quality of life, which may indicate avoidance of supposed public bodies. To counteract this, ASRs should receive general information about the German healthcare system. In addition, clear communication about the prospect of staying and the residence status is necessary to counteract uncertainties and the likely effects on mental and physical health.

Compared to a study analyzing the healthcare services utilization among asylum seekers who arrived in Germany in 2015, the prevalence of ASRs who visited a PCP at least once in the previous 3 months was lower in the current study (51% vs. 68%) [9]. Furthermore, the prevalence of hospitalization within 1 year was lower among ASRs in the current study than among refugees who arrived in Norway between 2005 and 2007 (14% vs. 37%) [10]. However, these differences may be at least partly due to the different study populations and time periods. In particular, the COVID-19 pandemic, which was declared in March 2020 by the WHO, may have had an impact on the healthcare services utilization of ASRs in Germany.

With regard to generalizability of the results of the current study, it can be stated that the Survey of Refugees, from which the sample was compiled, was designed to be representative of the population of ASRs who arrived in Germany between 2013 and 2016 [8].

As a major limitation of this study, it must be mentioned that due to an excessive amount of missing information in the sample of ASRs and due to a lack of consideration in the SOEP, potential further determinants of healthcare services utilization such as cultural barriers, previous health conditions, and trauma experiences were not available. Furthermore, potential confounders of healthcare services utilization such as regional healthcare policies, accessibility, and quality of care were not available for the analyses. Last, due to the unavailability of personal identifying data in the SOEP, it is not possible to link the self-reported data with actual healthcare records for cross-validation, so a self-reporting bias cannot be ruled out.

## Conclusion

This study was able to show minor associations between healthcare services utilization and flight-related characteristics of ASRs in Germany. In this regard, a feeling of being welcome and worries about not being able to stay in Germany may be determinants of healthcare services utilization with respect to the number of visits to PCPs and hospitalization. Further research is needed to investigate the associations between healthcare services utilization and flight-related characteristics of ASRs in larger samples, linked with actual healthcare records and over the long term.

## Supplementary Material

ckae135_Supplementary_Data

## Data Availability

The data can be applied via the website of the German Institute for Economic Research (DIW), Berlin. They are available free of charge for scientific, non-commercial use. The code used during the current study is available from the corresponding author on reasonable request for all interested researchers. Key points Not feeling welcome in Germany was associated with a higher utilization of PCPs and hospitalization among ASRs. Great worries about not being able to stay in Germany were associated with a lower utilization of PCPs among ASRs. ASRs should receive general information about the German healthcare system. The prospect of staying and the residence status should be clearly communicated with ASRs.

## References

[1] Statistisches Bundesamt. Bevölkerung Und Erwerbstätigkeit. Schutzsuchende Ergebnisse Des Ausländerzentralregisters 2020. Wiesbaden: Statistisches Bundesamt, 2021.

[2] Führer A. [Determinants of asylum seekers’ health and medical care in Germany]. Bundesgesundheitsblatt Gesundheitsforschung Gesundheitsschutz 2023;66:1083–91. 37707509 10.1007/s00103-023-03762-9 PMC10539189

[3] Norredam M , Nielsen SS, Krasnik A. Migrants' utilization of somatic healthcare services in Europe—a systematic review. Eur J Public Health 2010;20:555–63. 20040522 10.1093/eurpub/ckp195

[4] Grochtdreis T , König H-H, Dams J. Migration-related determinants of health-care service utilization among persons with a direct migration background in Germany: an exploratory study based on the German Socio-Economic Panel (SOEP). Eur J Health Econ 2024. 10.1007/s10198-024-01708-9 PMC11889003 39004694

[5] Andersen R , Newman JF. Societal and individual determinants of medical care utilization in the United States. Milbank Mem Fund Q Health Soc 1973;51:95–124. 4198894

[6] Klein J , von dem Knesebeck O. Inequalities in health care utilization among migrants and non-migrants in Germany: a systematic review. Int J Equity Health 2018;17:160. 30382861 10.1186/s12939-018-0876-z PMC6211605

[7] Kühne S , Jacobsen J, Kroh M. Sampling in times of high immigration: the survey process of the IAB-BAMF-SOEP survey of refugees. Survey Methods: Insights from the Field. 2019. https://surveyinsights.org/?p=11416.

[8] Grochtdreis T , König H-H, Dams J. Flight-related determinants of health-related quality of life of asylum seekers and refugees in Germany: a longitudinal study based on the German Socio-Economic Panel (SOEP). BMC Public Health 2024;24:1965. 39044148 10.1186/s12889-024-19489-4 PMC11264381

[9] Niedermaier A , Freiberg A, Tiller D et al Outpatient health care utilization and health expenditures of asylum seekers in Halle (Saale), Germany—an analysis of claims data. BMC Health Serv Res 2020;20:961. 33081775 10.1186/s12913-020-05811-4 PMC7576695

[10] Elstad JI. Register study of migrants’ hospitalization in Norway: World region origin, reason for migration, and length of stay. BMC Health Serv Res 2016;16:306. 27461121 10.1186/s12913-016-1561-9 PMC4962451

