# Supplementary material for: Flight-related determinants of healthcare services utilization of asylum seekers and refugees in Germany: a study based on the German Socio-Economic Panel
Source: Eur J Public Health. 2024 Aug 30;34(6):1173–6. doi: 10.1093/eurpub/ckae135 (PMC11631521; doi:10.1093/eurpub/ckae135)
Supplement: ckae135_Supplementary_Data [file ckae135_supplementary_data.docx]

**Table S1: Sociodemographic characteristics of the sample (n = 3134)**

| **Variables** | **N (%) / Mean (SE)** |
| --- | --- |
| Gender, female | 1620 (51.69) |
| Age, mean | 36.67 (0.20) |
| 18-24 | 477 (15.22) |
| 25-34 | 959 (30.60) |
| 35-44 | 968 (30.89) |
| ≥ 45 | 730 (23.29) |
| Country of birth |  |
| Syria | 1934 (61.71) |
| Iraq | 396 (12.64) |
| Afghanistan | 302 (9.64) |
| Europe | 65 (2.07) |
| Africa | 253 (8.07) |
| Other Asia^1^ | 184 (5.87) |
| School-leaving qualification^2^ |  |
| Secondary general school | 782 (24.95) |
| Secondary school | 775 (24.74) |
| Academic secondary school | 1403 (44.78) |
| No school-leaving qualification | 96 (3.05) |
| Employment, employed | 1973 (62.95) |
| Religious affiliation |  |
| Muslim | 2421 (77.26) |
| Other faith or non- denominational | 713 (22.74) |
| Oral German language skills |  |
| Very good | 343 (10.96) |
| Good | 1099 (35.07) |
| Not bad | 1181 (37.69) |
| Fairly bad | 453 (14.45) |
| Not at all | 57 (1.83) |
| Current health status |  |
| Very good | 1162 (37.08) |
| Good | 1191 (38.00) |
| Satisfactory | 462 (14.74) |
| Poor | 247 (7.88) |
| Bad | 72 (2.30) |
| Health-care services utilization |  |
| Number of visits to PCP in the previous three months, mean | 1.45 (0.10) |
| Hospitalization within one year | 430 (13.72) |

SE: Standard error; ^1^ Without Syria, Iraq and Afghanistan; ^2^ ‘Other school-leaving qualification’ is not shown

**Table 2: Flight-related characteristics of the sample (n = 3134)**

| **Variables** | **N (%) / Mean (SE)** |
| --- | --- |
| Economic situation in country of origin |  |
| Below average | 881 (28.12) |
| Average | 1509 (48.15) |
| Above average | 744 (23.73) |
| Connectedness with country of origin |  |
| (Very) strong | 1490 (47.54) |
| In some respects | 901 (28.76) |
| Hardly or not at all | 743 (23.70) |
| Reason for leaving country of origin^1^ |  |
| Fear of violent conflict/war, yes | 2578 (82.25) |
| Persecution, yes | 1490 (47.56) |
| Discrimination, yes | 1333 (42.54) |
| Living conditions, yes | 1237 (39.46) |
| Economic situation, yes | 957 (30.54) |
| Years since arrival at Germany, mean | 6.37 (0.91) |
| Residence status^2^ |  |
| No residence permission^3^ | 299 (9.55) |
| Temporary residence permission^4^ | 2653 (84.65) |
| Permanent residence permission^5^ | 138 (4.40) |
| Satisfaction with living situation |  |
| Dissatisfied | 528 (16.84) |
| Neither satisfied nor dissatisfied | 744 (23.74) |
| Satisfied | 1862 (59.42) |
| Discrimination due to origin |  |
| Often | 166 (5.28) |
| Rarely | 1009 (32.20) |
| Never | 1959 (62.51) |
| Feeling of missing people from the country of origin |  |
| (Very) often | 1959 (62.51) |
| Sometimes | 686 (21.90) |
| Rarely or never | 489 (15.59) |
| Feeling of being welcome in Germany |  |
| Fully or predominantly | 2670 (85.19) |
| In some respects | 342 (10.91) |
| Barely or never | 122 (3.90) |
| Worries of not being able to stay in Germany |  |
| Great worries | 928 (29.62) |
| Some worries | 663 (21.15) |
| No worries | 1543 (49.23) |
| Worries of not being able to return to country of origin |  |
| Great worries | 345 (11.00) |
| Some worries | 725 (23.13) |
| No worries | 2064 (65.87) |

SE: Standard error; ^1^ Multiple answers possible; ^2^ Another/no residence status not shown; ^3^ Permission to stay pursuant to Section 55 of the German Asylum Law (asylum seekers) or temporary suspension of deportation according to section 60a of the German Residence Act; ^4^Settlement permit according to Section 26 sub-section 3 of the German Residence Act; ^5^ Residence permit according to Section 25 sub-section 1 of the German Residence Act (persons entitled to asylum), according to Section 25 sub-section 2 of the German Residence Act (persons with refugee status), according to Section 22 or Section 23 of the German Residence Act (admission on humanitarian grounds), or residence permit pursuant to § 23a or § 25 sub-section 3, 4 or 5 of the German Residence Act (admission on other humanitarian grounds)
